# Protease-activated receptor-1 activation by granzyme B causes neurotoxicity that is augmented by interleukin-1β

**DOI:** 10.1186/s12974-017-0901-y

**Published:** 2017-06-27

**Authors:** Paul R. Lee, Tory P. Johnson, Sharmilee Gnanapavan, Gavin Giovannoni, Tongguang Wang, Joseph P. Steiner, Marie Medynets, Mark J. Vaal, Valerie Gartner, Avindra Nath

**Affiliations:** 1 Section of Infections of the Nervous System, National Institute of Neurological Disorders and Stroke, National Institutes of Health, 10 Center Drive, Building 10, Room CRC 3-2563, Bethesda, MD 20892 USA; 2 Centre for Neuroscience and Trauma, Blizard Institute, Barts and The London School of Medicine and Dentistry, London, UK; 3 Translational Neuroscience Center, National Institute of Neurological Disorders and Stroke, National Institutes of Health, Bethesda, MD 20892 USA; 4 Undiagnosed Diseases Program, National Human Genome Research Institute, National Institutes of Health, Bethesda, MD 20892 USA

**Keywords:** Neuroinflammation, Multiple sclerosis, Neurotoxicity, Protease-activated receptor, Granzyme B, Interleukin-1

## Abstract

**Background:**

The cause of neurodegeneration in progressive forms of multiple sclerosis is unknown. We investigated the impact of specific neuroinflammatory markers on human neurons to identify potential therapeutic targets for neuroprotection against chronic inflammation.

**Methods:**

Surface immunocytochemistry directly visualized protease-activated receptor-1 (PAR1) and interleukin-1 (IL-1) receptors on neurons in human postmortem cortex in patients with and without neuroinflammatory lesions. Viability of cultured neurons was determined after exposure to cerebrospinal fluid from patients with progressive multiple sclerosis or purified granzyme B and IL-1β. Inhibitors of PAR1 activation and of PAR1-associated second messenger signaling were used to elucidate a mechanism of neurotoxicity.

**Results:**

Immunohistochemistry of human post-mortem brain tissue demonstrated cells expressing higher amounts of PAR1 near and within subcortical lesions in patients with multiple sclerosis compared to control tissue. Human cerebrospinal fluid samples containing granzyme B and IL-1β were toxic to human neuronal cultures. Granzyme B was neurotoxic through activation of PAR1 and subsequently the phospholipase Cβ-IP3 second messenger system. Inhibition of PAR1 or IP3 prevented granzyme B toxicity. IL-1β enhanced granzyme B-mediated neurotoxicity by increasing PAR1 expression.

**Conclusions:**

Neurons within the inflamed central nervous system are imperiled because they express more PAR1 and are exposed to a neurotoxic combination of both granzyme B and IL-1β. The effects of these inflammatory mediators may be a contributing factor in the progressive brain atrophy associated with neuroinflammatory diseases. Knowledge of how exposure to IL-1β and granzyme B act synergistically to cause neuronal death yields potential novel neuroprotective treatments for neuroinflammatory diseases.

## Background

Multiple sclerosis (MS) is the most common disabling neuroinflammatory disease of young adults [1]. The pathophysiology of MS is not completely understood but involves chronic inflammation within the central nervous system [2] and progressive cerebral atrophy [3]. Symptomatic brain atrophy due to neurodegeneration appears early in the course of MS [4] and is progressive throughout the disease [5]. Grey matter loss is correlated with the amount of demyelination [6], but microscopically this atrophy is not associated with the robust infiltrative immune response of demyelination and must occur by another mechanism [7]. While the typical innate inflammatory process is well-described, there has not been a translation of this knowledge into effective treatments for the sequelae of neuroinflammatory diseases like MS. Given the significant association of neuronal loss with many disabling and refractory MS symptoms, the absence of proven interventions that provide neuroprotection in a setting of chronic inflammation represents a definite therapeutic gap.

A potential therapeutic target for neuroprotection in MS would be antagonism of receptors activated by cytotoxic substances released during inflammation. The protease-activated receptor (PAR) is a G-protein coupled receptor whose unique activation requires cleavage of an extracellular sequence by a protease, a family of enzymes including many inflammatory mediators [8]. There are four known PAR subtypes; all have been found on neurons throughout the brains of several species [8]. The PAR subtype designated “PAR1” was initially identified as the native receptor for thrombin [9], but it is now known that neuronal PARs can be activated by other proteases capable of cleavage within the extracellular sequence, including granzyme B [10]. The function of neuronal PAR1 appears varied. Neuronal PARs are activated by proteases secreted by neurons [11] and by proteases that breech the blood-brain barrier [12]. During development, PAR1 activation is necessary for neuronal process extension and survival [13, 14]. Neurons lacking PAR1 cannot exhibit long-term potentiation [15]. In mature neurons, PAR1 agonists are neurotoxic [16, 17]. Our laboratory has demonstrated that a single exposure to granzyme B released by T lymphocytes is neurotoxic through PAR1 activation [10, 18].

IL-1β upregulates neuronal PAR1 expression [16]. IL-1β circulating within the cerebrospinal fluid (CSF) is a biomarker of neuroinflammation caused by MS [19] and subacute sclerosing panencephalitis [20]. IL-1β augments neuronal excitotoxicity [21]. Increased levels of IL-1β in the CSF of patients with MS correlate with greater cortical atrophy [22].

Prior in vitro investigations of granzymes and interleukins in MS have examined single-dose exposures, not chronic effects. In neuroinflammation, significant elevations of IL-1β and granzyme B have been documented within the CSF [18, 23], and therefore, neurons in vivo reside within a milieu providing continuous exposure to these neurotoxic entities.

In this study, we found that PAR1 is increased on neurons’ surfaces within the brains and lesions of patients with MS. Further, we demonstrated a unique mechanism of how chronic PAR1 activation by granzyme B is neurotoxic and determined that this specific mechanism could be driven by IL-1β. We sought to expand the role of granzyme B in neuronal death and provide a more thorough explanation of how chronic PAR1 activation leads to neurotoxicity. These findings identify several novel targets for neuroprotective treatments.

## Methods

### Human tissue samples

Histochemical portions of this study were performed on formalin-fixed, paraffin-embedded archival material of eight (*n =* 4 patients with MS, *n =* 4 control) human brain autopsy tissue samples obtained from The Human Brain and Spinal Fluid Resource Center, University of California Los Angeles, CA. In the four subjects designated with a diagnosis of MS, all patients had a chronic course. The four patients serving as controls in histochemistry experiments did not have neurological diseases (Table 1).

**Table 1:** Clinical and demographic features of patients included in immunohistochemical studies

Diagnosis	Gender	Age at death	Postmortem interval	Additional diagnoses
MS	F	61	6.8 h	Lung cancer
MS	F	65	14.2 h	Depression
MS	F	46	13.0 h	Cervical cancer
MS	M	57	24.8 h	Myocardial infarction
Control	F	62	Unknown	Otosclerosis
Control	F	87	Unknown	None
Control	F	79	1.0 h	Bronchiectasis, Torticollis
Control	M	85	Unknown	None

For neurotoxicity and enzyme-linked immunosorbant assay studies, human CSF samples were obtained via lumbar puncture from patients diagnosed with secondary progressive MS (*n =* 7) involved in a clinical trial of lamotrigine as described previously [24, 25] or from NINDS patients without neuroinflammation (*n =* 7) who served as controls.

The Office of Human Subjects Protection and Research at the National Institutes of Health approved this research.

### Neuronal cultures

iCell neurons (Cellular Dynamics International, Madison, WI) which were derived from induced pluripotent stem cells were used for the viability and toxicity studies. Neurons were cultured according to the manufacturer’s instructions. In brief, 96-well plates were coated with 100 μl of 0.01% (*w*/*v*) poly-L-ornithine (Sigma-Aldrich, St. Louis, MO) for 1 h at room temperature, washed twice with sterile distilled water, and finally coated with a 3.3-μg/mL solution of laminin (Sigma-Aldrich) and molecular biology-grade water. The dishes with laminin solution were incubated in a 37 °C incubator for at least 1 h prior to plating of neurons. The laminin solution was aspirated immediately before addition of the cell suspension. A vial containing 2.5 million iCell neurons was removed from liquid nitrogen storage and immediately placed in a 37 °C water bath for exactly 3 min. The cells were transferred to a 50-ml conical tube and diluted to 10 ml total volume with iCell Complete Maintenance Media (Cellular Dynamics International) dropwise per manufacturer’s protocol. Contents of the flask were swirled gently throughout addition of media. Cell viability was assessed from the initial volume and then further dilution was performed to ensure that sufficient quantity of cell suspension was added to 96-well plates to ensure at least 80,000 viable cells were seeded per square centimeter. The cells were kept in a 37 °C incubator in 5% (*v*/*v*) CO_2_. One day after initial plating, there was a 100% exchange of fresh iCell Complete Maintenance Media, and this media was changed (50–75% exchange of the total volume) every 1–2 days to ensure maximum viability prior to experimental use. Neurons were not used for experiments until at least 3 days after successful plating. Opti-MEM reduced-serum media (Life Technologies, Carlsbad, CA) supplemented with 2% (*v*/*v*) B27 (Life Technologies) served as the control and treatment media for all cultures. For multiple day experiments, there was a 100% replacement of media and any additional components every 24 h until analysis. If an additive such as DMSO was used to promote solubility of a compound being evaluated, then an equivalent amount of the additive was added to control media daily. In experiments using human CSF, the cultures were maintained in a 100-μl volume of undiluted thawed CSF from a single donor with a 100% daily exchange to replenish CSF and any additives such as protease inhibitors or interleukin-1 (IL-1) receptor antagonist. Recombinant human granzyme B, IL-1β, and thrombin as well as agonists/antagonists of PAR1 and second messenger components were obtained from R&D Systems/Tocris Bioscience (Minneapolis, MN). Any agonists, antagonists, or inhibitors were added at least 30 min prior to addition of granzyme B and IL-1β. Atopaxar (E5555) was prepared using a variant of the convergent synthesis described in the patent # US 2006/0058370 A1 [26]. The coupling of a fluorinated cyclic benzamidine (A) with a morpholine-substituted phenacyl bromide (B) in THF gave the HBr salt of Atopaxar as seen below.
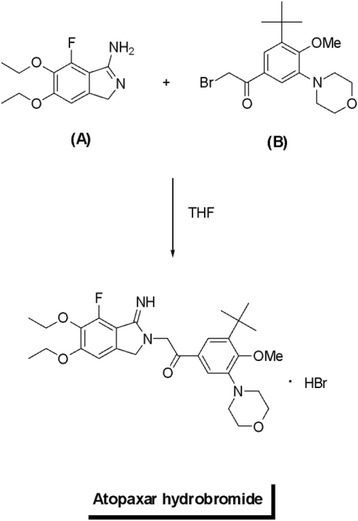


**Scheme 1:**
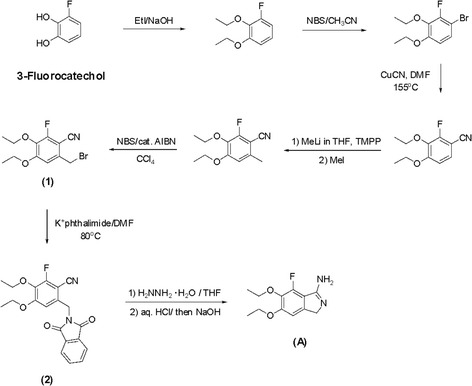
Synthesis of florinated cyclic benzamidine

**Scheme 2:**
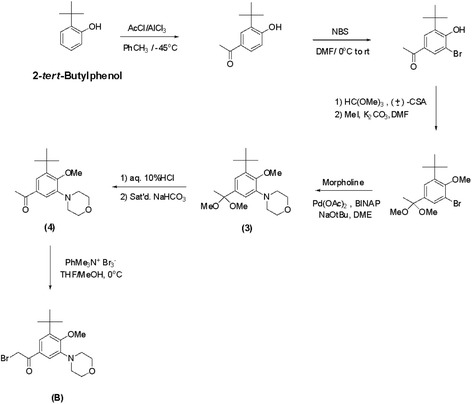
Synthesis of morpholine-substituted phenacyl bromide

The syntheses of (A) and (B) are shown in the following Schemes 1 and 2.

Starting with 3-fluorocatechol, the synthesis required essentially seven steps. Benzamidine (A) was ultimately prepared from phthalimide (2) which could be prepared from bromide (1).

Starting with 2-*tert*-butylphenol, the synthesis of (B) required essentially *six* steps. One slight variation from the Eisai patent procedure involved the hydrolysis of ketal (3) back to the methyl ketone (4). This material could be purified chromatographically before brominating, resulting in much cleaner phenacyl bromide (B). Atopaxar hydrobromide prepared in this manner gave satisfactory NMR, elemental analysis, and liquid chromatography/mass spectroscopy. The crystal retained 0.25 equivalent of THF as seen by nuclear magnetic resonance and elemental analysis.

### Immunohistochemistry

Sections of 100-μm thickness were prepared from formalin-fixed and paraffin-embedded brain sections from control and from subcortical white matter containing known lesions in patients with MS. They were deparaffinized using standard protocols. Antigen retrieval was performed using sodium citrate buffer (10 mM sodium citrate, 0.05% (*v*/*v*) Tween 20). Endogenous peroxidase was inhibited using a dual enzyme block solution (Dako, Carpinteria, CA) and native protein block was achieved with a serum-free protein block solution (Dako). Following a TBS-Tris wash, sections were immunolabeled for 1 h at room temperature using mouse monoclonal anti-PAR1 (1:500, Abcam, San Francisco, CA) or rabbit polyclonal anti-IL1 receptor (1:100, Abcam) as primary antibodies. After a wash with TBS-Tris, sections were incubated with a Powervision poly-HRP anti-rabbit or anti-mouse secondary antibody solution (Dako). Development for visualization of antibody binding within the tissue was achieved using the chromagen 3,3'-diaminobenzidine (Sigma). Hematoxylin (10%; *w*/*v*) was used to provide a counterstain. Omissions of the primary or secondary antibodies were used as negative controls.

### Quantification of immunohistochemistry

The total number of cells and immunolabeled cells were counted manually in a minimum of ten fields from within and immediately around subcortical white matter (healthy or containing a lesion) and the adjacent cerebral cortex (layer VI) of patients with MS and controls. Lesions were defined based on demyelination borders identified with Luxol Fast Blue staining of an adjacent section. For quantification of cell number within a tissue section, a 20-μm grid was aligned manually across the desired counting area and all cells fully within the section were counted using a microscope with a 20× objective configured to image in transmitted light mode. Optimal light and focal settings were established prior to counting. Serial sections stained with anti-NeuN antibody (1:1000, Abcam) were used to definitively identify whether a given counted cell nucleus belonged to a neuron. Cells were graded as “no,” “low”, or “high” expression based on comparative visual appearance. Regions with significant tissue artifacts or overlapping with a tissue edge were excluded from analysis. The total density of cells was estimated from the observed counts using a modified Abercrombie estimation [27] and is expressed as a calculated density (neurons/mm^2^).

### Enzyme-linked immunosorbent assay

Concentrations of granzyme B (eBioscience, San Diego, CA) and IL-1β (Abcam) were quantitated in CSF samples using enzyme-linked immunosorbent assay (ELISA) kits according to manufacturer’s instructions. CSF samples from patients with MS or control patients were incubated on microtiter strips with biotinylated monoclonal anti-granzyme B or IL-1β antibody. Streptavidin-peroxidase and substrate were added, and absorbance was measured at 450 nm. Quantitation of secreted granzyme B and IL-1β were determined based on a standard curve generated from known standards included in the kits.

### Other CSF assays

Total protein concentration in CSF samples was analyzed using a BCA Protein Assay (Life Technologies) following the manufacturer’s instructions. Glucose concentration was estimated in CSF using a Glucose Colorimetric Assay Kit (Cayman Chemical, Ann Arbor, MI). A glutamate assay kit (Abcam) was used to measure CSF glutamate concentrations.

### Neuronal viability and toxicity

The MultiTox-Fluor Multiplex Cytotoxicity Assay (Promega, Madison, WI) was used for simultaneous evaluation of viability and toxicity in neuronal cultures for all conditions. Cultures were maintained in 96-well plates with black walls in a total volume of 100 μl of media with or without experimental additives such as granzyme B, IL-1β, or inhibitors. To inhibit granzyme B activity in CSF samples, these samples were pretreated with the selective cell-permeable protease inhibitor Z-Ala-Ala-Asp-chloromethylketone (Enzo Life Sciences, Farmingdale, NY). The optimal concentration for additives was determined empirically. The MultiTox-Fluor Multiplex Cytotoxicity Assay Reagent was prepared at two times concentration according to the manufacturer’s instructions and 100 μl of this assay reagent was added to each well. Each well’s contents were mixed by gentle pipetting. The plates were returned to the incubator for 1 h. Fluorescence intensity was then measured using a FlexStation 3 Microplate Reader and SoftMax Pro software (Sunnyvale, CA) with the following settings, for viability: excitation = 400 nm and emission = 505 nm and for toxicity: excitation = 485 nm and emission = 520 nm. All conditions were performed at least in duplicate and data were pooled from multiple independent experiments. For multiple day cumulative toxicity measurements, several wells were analyzed at each daily time point to generate a cumulative toxicity percentage for a given day. Data were collected as relative fluorescence units. Conditions included in each experiment included empty wells as well as wells without neurons containing just media and additives for background assessment, wells containing neurons in experimental media with appropriate additives which served as negative controls, and wells with sufficient saponin 0.1% (*w*/*v*) added prior to assessment to ensure 100% toxicity which served as the positive control condition for toxicity calculation purposes.

### Immunocytochemistry

Neurons were fixed in 4% (*w*/*v*) paraformaldehyde for 10 min at room temperature, followed by three washes with PBS. After incubation in 0.1% (*v*/*v*) Triton X-100 in PBS (PBS-T) for 10 min at room temperature, the cells were incubated with blocking buffer (PBS-T containing 4% (*v*/*v*) goat serum and 1% (*v*/*v*) glycerol (Sigma-Aldrich) at room temperature for 20 min. Cells were immunostained with mouse monoclonal anti-PAR1 (1:500, Abcam) and rabbit polyclonal anti-IL1 receptor (1:100 Abcam), followed by corresponding secondary antibodies (anti-mouse Alexa Fluor 488 1:200 Abcam, anti-rabbit Alexa Fluor 647 1:200 Abcam) and 4′,6-diamidino-2-phenylindole (DAPI) nuclear staining. Images were acquired on a Zeiss LSM 510 META multiphoton confocal system (Carl Zeiss, Dublin, CA) or an EVOS fluorescence microscope (AMG, Bothell, WA).

### Immunocytochemistry quantification

The total number of DAPI-stained nuclei per field provided an estimate of the total number of neurons present. Degenerating nuclei were excluded from counts as were indistinct DAPI-positive structures. The number of PAR1-positive neurons was then counted after overlaying these images on the DAPI-positive images. Only neurons with distinct DAPI-positive nuclei were noted for analysis. The percentage of positive neurons for a given field was generated by dividing the number of PAR-stained neurons by the total of the counted DAPI-positive nuclei.

### Real-time quantitative polymerase chain reaction

PAR1 expression was determined by quantitative real-time polymerase chain reaction (qPCR) as described elsewhere [28]. Briefly, cultured iCell neurons were treated with granzyme B, interleukin 1β, and/or siRNA designed to suppress PAR1 for the stated times and total RNA was extracted using a Qiagen RNeasy mini kit (Qiagen, Valencia, CA). The RNA samples were treated with DNase I (Invitrogen, Grand Island, NY) for 15 min followed by Turbo DNAse treatment (Life Technologies) according to the manufacturer’s instructions for “stringent” conditions. Then complementary DNA (cDNA) was synthesized from this purified total RNA using random primers (PAR1 sense: 5′-AGTAGGCTATTCCTGAGAGCTGCAT-3′; PAR1 antisense: 5′-ATGGCCCTGGCATGTGTCT-3′) and SuperScript III First-Strand cDNA kit (Invitrogen). The resulting cDNA was then mixed with gene-specific primers and SYBR Green PCR universal master mix (Life Technologies). RNA was amplified using an ABI Prism 7000 Sequence Detection System (Applied Biosystems, Foster City, CA) according to the manufacturer’s instructions. The relative levels of messenger RNA (mRNA) were calculated using the ΔΔCt method by normalization to an internal control, glyceraldehyde-3-phosphate dehydrogenase (GAPDH).

### siRNA

The Accell siRNA kit (Thermo Scientific, Waltham, MA) was used to reduce expression of PAR1. A pooled set of siRNA sequences, three directed against the human F2R (thrombin receptor, PAR1) Open Reading Frame and one against the 3′UTR of the gene were used. The siRNA introduction was performed according to the manufacturer’s instructions for “plated cells sensitive to media-free delivery conditions.” To maximize siRNA delivery to neurons, an aliquot of *Trans*IT-*X*2 Dynamic Delivery System (Mirus Bio, Madison, WI) was added with the diluted siRNA to the Accell delivery buffer. After 72 h, the siRNA solution was replaced with experimental media following a wash with PBS. Reduction in PAR1 protein was confirmed by a Western blot analysis using anti-PAR1 antibody (1:1000) following a methodology otherwise as described elsewhere [18].

### Statistics

Statistical analyses were performed using Graphpad Prism (La Jolla, CA) software (version 6.05). For toxicity and viability measures, raw data were compiled into percentage of the controls used in their respective experiments. Data are presented as mean ± standard error of the mean. Data analysis included an analysis of variance (ANOVA; one-way, two-way, or repeated measures as indicated) with a post hoc test to correct for multiple comparisons as noted, a Tukey test was used when comparisons of all means were made to one another, a Dunnett test was used for comparisons of means to a single appropriate control condition. Correlations were used to generate Pearson statistics where indicated. Statistical significance was established as a *p* value <0.05.

## Results

### PAR1 surface expression is increased in brain tissue from patients with MS

Human post-mortem brain tissue sections from patients with MS and control patients lacking neuroinflammatory disease were used to investigate whether there were qualitative or quantitative differences in neurons expressing PAR1 and the IL-1 receptor within healthy and inflammatory subcortical tissue samples.

**Fig. 1:**
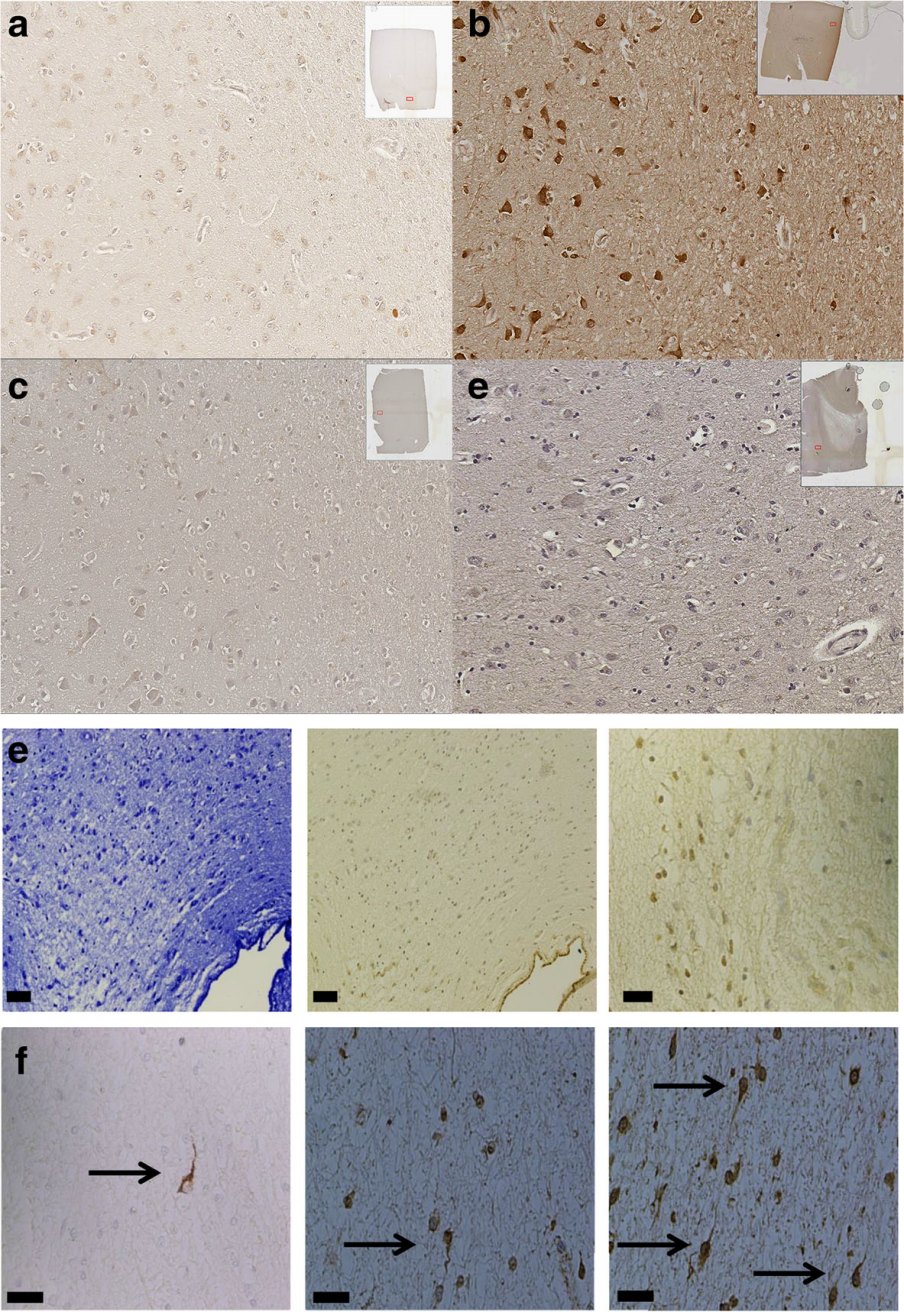
PAR1 and IL-1 receptor surface expression in human brain tissue. **a** Immunohistochemistry for PAR1 demonstrates a low level of baseline staining in healthy human brain tissue. **b** There is more prominent PAR1 staining within MS brain tissue. **c** IL-1 receptor staining in healthy tissue and **d** around a demyelinated plaque were similar. **e** Luxol fast blue stain shows a demyelinated region and subsequent images show PAR1 staining is pronounced within a lesion’s borders and follows a gradient diminishing at approach to the lesion’s outermost border. **f** Within demyelinated lesions there are darkly stained PAR1-positive neurons with visible unmyelinated axons (*arrows*). Bars = 50 μm

Brain tissue from both control patients and patients with MS exhibited PAR1 staining; neurons and glia had detectable surface expression of PAR1 (Fig. 1a, b). IL-1 receptor staining was consistently rated as “low” or “no” expression without visual distinction between tissue from patients with MS or control patients (Fig 1c, d). Brain tissue originating from patients with MS had significantly fewer neurons overall owing to loss of cellularity within white matter lesions and in the cortex (Table 2). Despite the discrepancy in numbers of cells, there were approximately 30% more PAR1 high expression neurons within the cortex adjacent to, inside, and around lesions from brain tissue samples of patients with MS compared to healthy patients’ tissue. Regions around and within demyelinated lesions had concentrations of “high expression” rated cells (Fig. 1e). Additionally, there were prominent PAR1-positive neurons with darkly stained axons situated inside demyelinating lesions (Fig. 1f); similar neurons were not found in healthy control samples. Omitting the primary antibodies from the staining protocol yielded no visible PAR1 or IL-1 receptor staining in brain tissues (not shown).

**Table 2:** Neuronal density in brain tissue from patients with MS or control patients

Density (neurons/mm^2^)	Control cortex	MS cortex	MS lesion	Significance
Total number	65.3 ± 3.9	55.6 ± 2.1*	46.6 ± 8.2**	*p < 0.01, ** *p* < 0.001
PAR1 high expression	29.0 ± 4.2	37.7 ± 2.8*	44.3 ± 6.8*	*p < 0.01
IL-1 receptor low expression	58.9 ± 1.1	59.2 ± 1.3	58.1 ± 1.4	NS

Data are mean ± standard error of the mean

### Inflammatory CSF is neurotoxic via granzyme B

We used ELISA kits to determine the levels of granzyme B and IL-1β within samples of human CSF to confirm the relative amounts of granzyme B and IL-1β in CSF samples from patients with MS. Granzyme B levels in CSF from patients with MS ranged from 10.11 to 12.22 pg/ml (mean = 14.22 pg/ml), and the IL-1β levels were detectable at values between 7.30 and 24.56 pg/ml (mean = 17.38 pg/ml). There was a significant positive correlation between granzyme B and IL-1β within CSF samples from patients with MS (*r =* 0.852, *p* < 0.015). In control samples, the granzyme B levels were near the assay’s detection threshold (range <0.20–1.10 pg/ml) and IL-1β levels in CSF samples from control patients were below the assay’s detection threshold (<6.5 pg/ml).

**Fig. 2:**
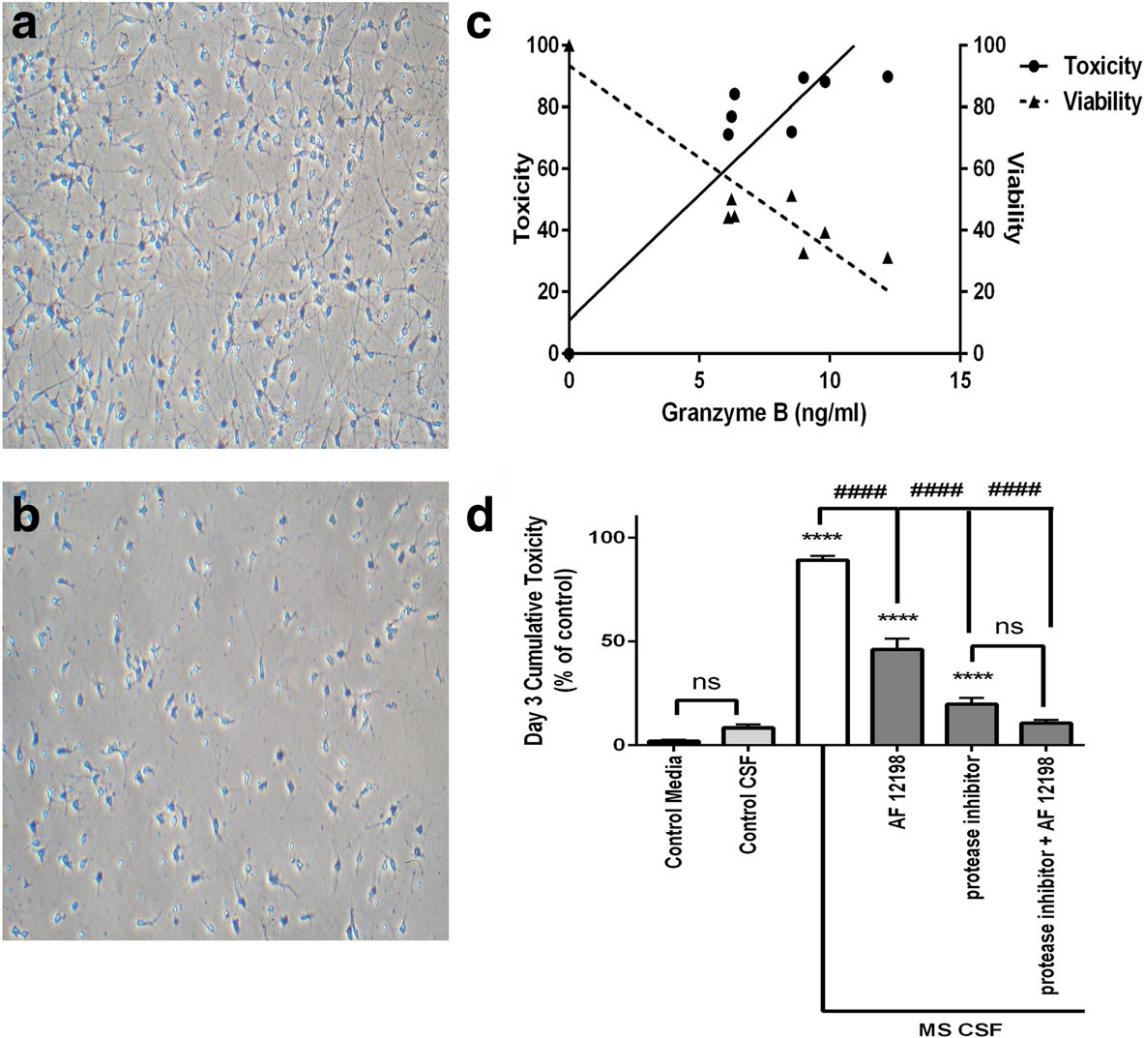
Effects of cerebrospinal fluid (CSF) samples from patients with MS on human neuronal cultures. Cortical neurons derived from induced pluripotent stem cells were treated with undiluted human CSF from patients with MS or from patients without neuroinflammatory diseases for 3 days. **a** Neuron cultures maintained in CSF from control patients verified to have low levels of granzyme B and interleukin-1β had no evidence of injury or toxicity. **b** Neuron cultures exposed to CSF from patients with MS were fewer in number and had morphological evidence of damage (i.e., shorter axons, simplified arborization). **c** The toxicity induced by CSF from patients with MS was directly related to the CSF’s granzyme B content. Conversely, neuronal viability diminished with increasing concentration of granzyme B within a given sample. Data are shown as the mean from duplicate experiments. **d** The neurotoxicity of CSF from patients with MS was significantly greater at 3 days than CSF from control patients and could be abolished by the cell-permeable granzyme B inhibitor Z-Ala-Ala-Asp-chloromethylketone (25 μM) and partially prevented by inhibition of the interleukin-1 receptor using the antagonist AF 12198 (1 μM). A mixture of granzyme B inhibitor and AF 12198 was no more effective than the granzyme B inhibitor alone at preventing cumulative neurotoxicity. ****p < 0.0001 relative to CSF from control patients, ####*p* < 0.001 relative to CSF from patients with MS, one-way ANOVA with post hoc Tukey’s test, *n =* 7 per condition. Data are shown as the mean ± standard error of the mean from duplicate experiments

To assess whether CSF from patients with MS was neurotoxic, aliquots of the assayed CSF samples were then introduced into human neuron cultures derived from induced pluripotent stem cells for 3 days. Viability and toxicity were measured simultaneously within each sample well at the end of the third day. Following 3 days of CSF exposure, there was significant neurotoxicity (>70%) in cultures cultured with CSF from patients with MS compared with neurons in control media (Fig. 2a, b). When viability and toxicity were analyzed for the samples, there was a significant negative correlation (*r* ^2^ = 0.882, *p* < 0.002) between the concentration of granzyme B within the sample well and the viability of the neurons within the well after 3 days (Fig. 2c). A reciprocal significant positive correlation (*r* ^2^ = 0.853, *p* < 0.0004) between granzyme B concentration and toxicity was also observed (Fig. 2c). The correlations between IL-1β concentration and toxicity (*r* ^2^ = 0.5759, *p* < 0.01) and viability (*r* ^2^ = 0.5932, *p* < 0.009) were significant but less robust. The control patients’ CSF was as non-toxic to the neuronal culture as the control neuronal media (Fig. 2d). There was no difference between the CSF samples from patients with MS and control CSF with respect to concentrations of glucose, total protein, or L-glutamate (Table 3).

**Table 3:** Cerebrospinal fluid parameters

	Control	Multiple sclerosis	Significance
Glucose (mg/dl)	75.43 ± 7.50	73.14 ± 7.12	NS
Total protein (mg/dl)	19.86 ± 2.30	20.57 ± 3.84	NS
Glutamate (μg/l)	0.28 ± 0.11	0.21 ± 0.04	NS
Granzyme B (pg/ml)	0.05 ± 0.02	14.22 ± 1.49	*p* < 0.05
Interleukin-1β (pg/ml)	<6.5	17.38 ± 2.17	*p* < 0.05

Data are mean ± standard error of the mean

When CSF from patients with MS was pretreated with a granzyme B-specific protease inhibitor prior to being added into neuronal cultures, the observed neurotoxicity was reduced and not significantly different than control media with protease inhibitor. Addition of a selective IL-1 receptor antagonist (AF 12198) decreased toxicity by approximately 50%. Protease inhibition together with IL-1 receptor antagonism was as effective as protease inhibition alone in preventing neurotoxicity after 3 days of exposure (Fig. 2d).

### Granzyme B and IL-1β act together to diminish neuronal viability

**Fig. 3:**
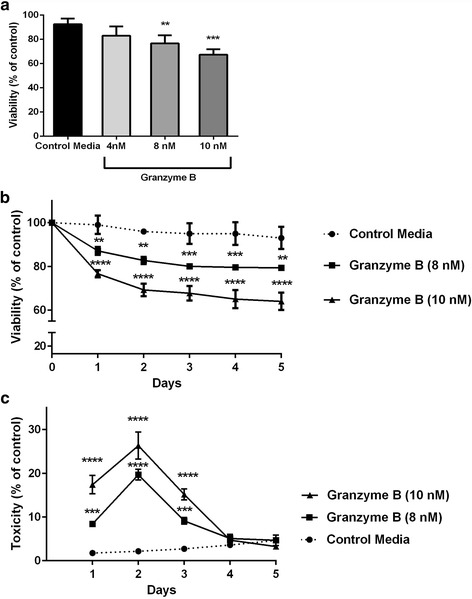
Granzyme B-mediated neurotoxicity. **a** Pure cortical neuron cultures were treated with granzyme B and the viability and toxicity were measured simultaneously. A single, 24-h exposure to granzyme B at escalating concentrations (4, 8, and 10 nM) replicated a dose-dependent decrease in neuron viability. The 8- and 10-nM doses caused significantly decreased viability relative to control media. **p < 0.01 and ***p < 0.001, respectively, one-way ANOVA with post hoc Dunnett test, *n =* 8 per condition. Data are shown as the mean ± standard error of the mean from four separate experiments. **b** When granzyme B was added for five consecutive days, the viability of the all cultures stabilized at day 3. **p < 0.01, ***p < 0.001 and ****p < 0.0001 relative to control media, repeated measures ANOVA with post hoc Dunnett test, *n =* 10 per condition. Data are shown as the mean ± standard error of the mean from three separate experiments. **c** Granzyme B-mediated toxicity peaked on day 2 of exposure followed by a decline to control toxicity levels on day 4. ***p < 0.001 and ****p < 0.0001 relative to control media, repeated measures ANOVA post hoc Dunnett test, *n =* 10 per condition. Data are shown as the mean ± standard error of the mean from three separate experiments

To determine the effect of repeated chronic exposure of granzyme B on neurons, human neurons were plated at a known, fixed concentration and allowed to mature per manufacturer’s guidelines. Once neurons exhibited a mature phenotype, they were exposed to a range of concentrations of granzyme B for 7 days. To maintain potency throughout the duration of the experiments, granzyme B was replenished on a daily basis for 7 days with neuronal viability measured in a set of neurons reared in parallel at each 24-h time point. As in previous studies, a single 24-h exposure to granzyme B yielded a dose-dependent reduction in viability (Fig. 3a). However, when granzyme B was replenished on a daily basis, the effect of granzyme B in culture reached a plateau (Fig. 3b). By day 4, the granzyme B toxicity approached zero, and the toxicity converged on that of the control conditions and subsequent days’ exposures were not toxic relative to control media (Fig. 3c). The decline in viability noted on day 5 in culture was universal and is an expected outcome from depriving these neurons of proprietary media’s nutrients.

**Fig. 4:**
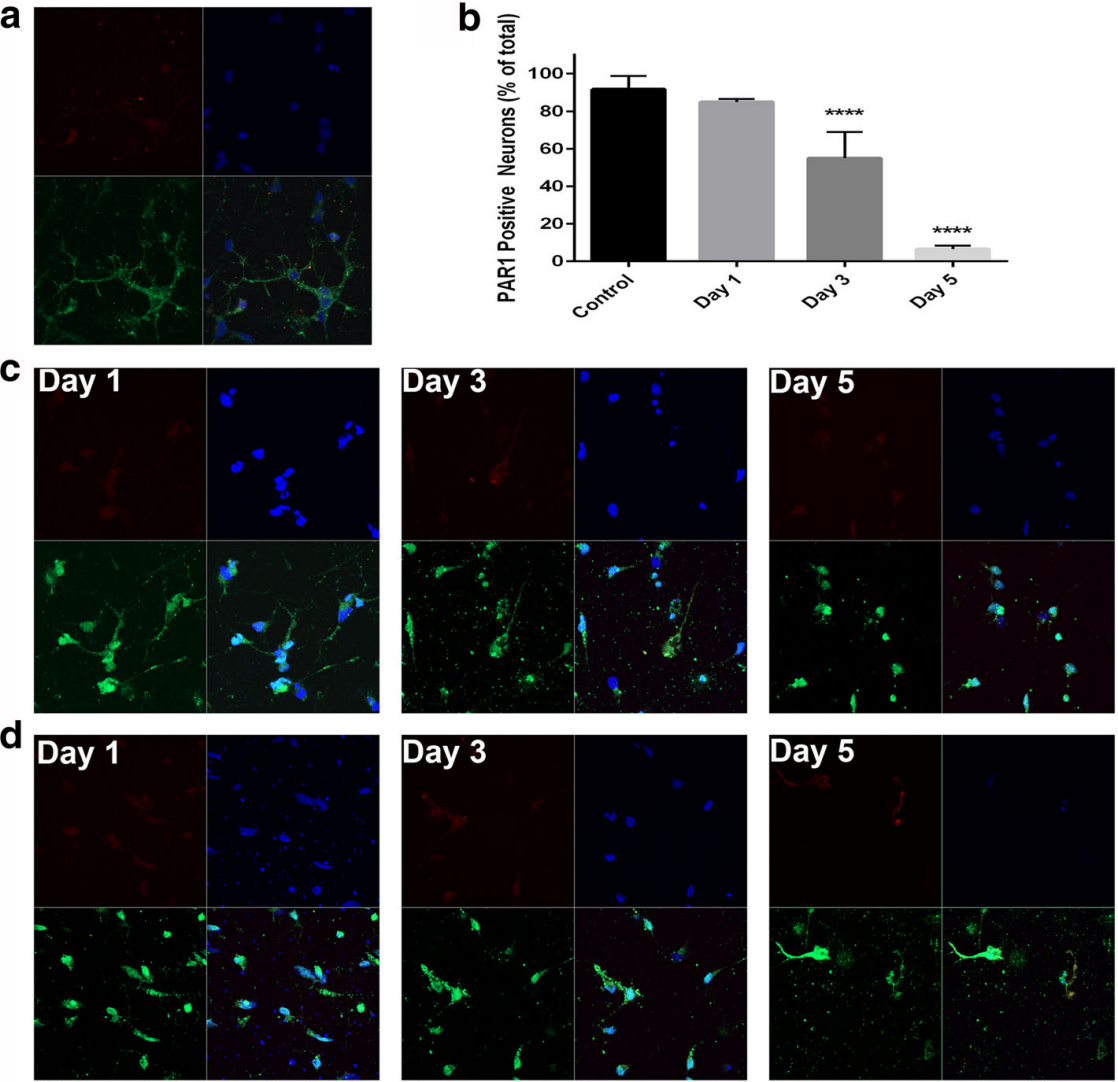
PAR1 and IL-1 receptor surface expression on cultured neurons. **a** At baseline on day 0, most neurons showed surface expression of PAR1 by immunocytochemistry visualized here using a green fluorescent secondary antibody (*bottom left panels*). A red fluorescent antibody was used to visualize IL-1 receptor (*top left panels*), and IL-1 receptor surface expression was noted on all neurons and was not significantly changed during granzyme B exposure. Nuclei were stained with DAPI (*blue*) and superimposed onto the combined image (*lower right panels*). **b** The percentage of neurons with PAR1 surface expression visible by fluorescence significantly decreased over the course of 5 days of chronic exposure to granzyme B and IL-1β relative to the total number of DAPI-stained nuclei at any given time ****p < 0.0001 relative to control media, repeated measures ANOVA with post hoc Dunnett test, *n =* 8 per condition. Data are shown as mean ± standard error of the mean from four separate experiments. **c** On days 1, 3, and 5 in media containing granzyme B (10 nM), there was a reduction in the number of neurons in culture evident at all time points and a morphological changes consistent with chronic injury including shortened axons. **d** After 1, 3, and 5 days in media containing granzyme B (10 nM) and IL-1β (20 ng/ml), the total number of neurons in culture, and the number of neurons staining positive for PAR1 were both significantly reduced

To investigate the basis for the observed stabilization in viability and toxicity, immunocytochemistry techniques were used to visualize the PAR1 receptor on the surface of cultured neurons, and we observed that consistently the overwhelming majority of neurons (91.66 ± 4.09%) in this specific neuron preparation had visible surface expression of PAR1 at baseline (Fig. 4a). Following several days of granzyme B exposure when toxicity returned to control media equivalence, we observed that the percentage of PAR1-positive neurons would sequentially decline to less than 10% (6.57 ± 0.64%) of the total number (Fig. 4b). The expression of IL-1 receptor increased after initial granzyme B exposure then remained stable for the duration of exposure (Fig. 4c, d). The timing of the decline in the percentage of PAR1-positive neurons in these cultures mirrors the fall in toxicity we observed with repeated presentation of granzyme B (Fig. 4e). Thus, it appeared that a finite number of neurons expressing PAR1 were at-risk to granzyme B and the ultimate total toxicity achieved corresponded to neurons expressing high levels of PAR1 on their surfaces.

**Fig. 5:**
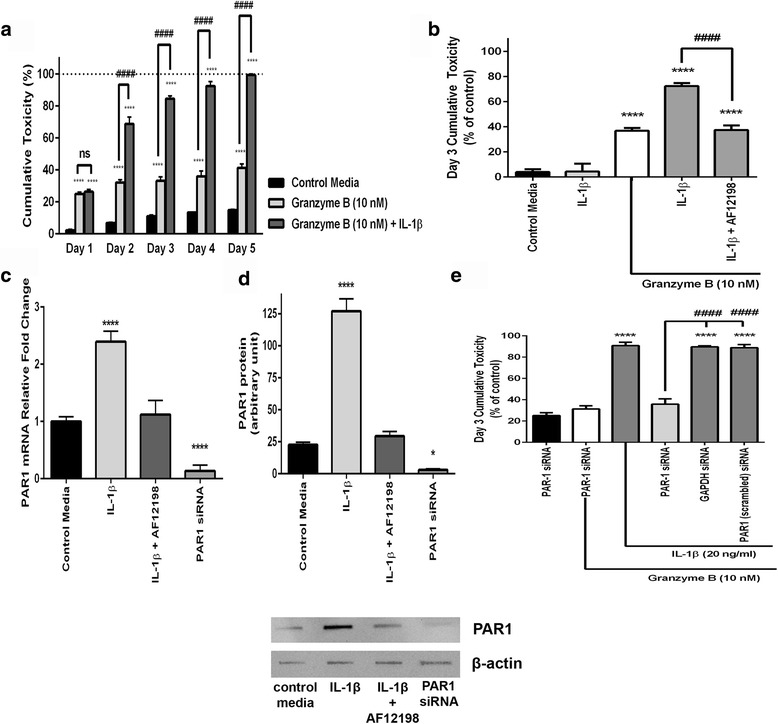
Enhancement of granzyme B toxicity with IL-1β. **a** IL-1β (20 ng/ml) augments granzyme B neurotoxicity. Cumulative neurotoxicity of combined granzyme B and IL-1β reaches 100%. ****p < 0.0001 cumulative toxicity relative to control media, ####*p* < 0.0001 cumulative toxicity relative to granzyme B + IL-1β (two-way ANOVA with post hoc Tukey’s test, *n =* 5/condition. Data are mean ± standard error of the mean from three separate experiments). **b** IL-1β is not toxic by itself. The IL-1 receptor antagonist AF 12198 (1 μM) abolished IL-1β effect after 3 days of co-culture with granzyme B and IL-1β (20 ng/ml). ****p < 0.0001 relative to control media, ####*p* < 0.0001 between granzyme B + IL-1β and granzyme B+ IL-1β + IL-1 receptor antagonist, repeated measures ANOVA with post hoc Tukey’s test, *n =* 6/condition. Data are mean ± standard error of the mean from three separate experiments. **c** After 3 days of exposure, IL-1β (20 ng/ml) significantly increased PAR1 mRNA as shown by qPCR and PAR1 protein relative to control levels using Western blot analysis. The IL-1 receptor antagonist AF 12198 (1 μM) blocked the IL-1β effect on PAR1 mRNA and protein. A pooled PAR1 siRNA preparation reduced PAR1 mRNA and protein below control conditions. **d** Western blot analysis (*below bar graph*) confirmed the relative amounts of PAR1 protein in respective conditions. *p < 0.05, ****p < 0.0001 relative to control media, one-way ANOVA with post hoc Tukey’s test, *n =* 4/condition. Data are mean ± standard error of the mean from duplicate experiments. **e** Granzyme B had no toxicity when PAR1 siRNA reduced PAR1 protein levels. A pooled PAR1 siRNA preparation inhibited all granzyme B neurotoxicity; IL-1β had no effect. Two control siRNA, anti-GAPDH, and a scrambled PAR1 sequence had no effect. ****p < 0.0001 relative to control media, ####*p* < 0.0001 between PAR1 siRNA + granzyme B+ IL-1β and other siRNA as noted, one-way ANOVA with post hoc Tukey’s test, *n =* 8/condition. Data are mean ± standard error of the mean from four separate experiments

We explored the role of neuronal surface PAR1 in granzyme B neurotoxicity PAR1 by both increasing and decreasing PAR1 expression. IL-1β increases PAR1 expression on neurons, and administering IL-1β with granzyme B had a dramatic effect on neurotoxicity by sustaining neural losses throughout the 5-day observation such that the cumulative loss of neurons by day 5 reached approximately 100% (Fig. 5a). The increased toxicity was not due to a direct toxic effect of IL-1β because IL-1β was not toxic when administered alone (Fig. 5b). We introduced a selective antagonist of the IL-1 type I receptor (AF12198) to block IL-1β signaling. The antagonist blocked the IL-1β component of granzyme B toxicity (Fig. 5b). IL-1β acting at the IL-1 receptor altered PAR1 production in the neurons. IL-1β caused PAR1 mRNA expression to increase twofold and PAR1 protein to increase fourfold (Fig. 5c). Antagonism of IL-1 receptors prevented a significant IL-1β-mediated increase in PAR1 mRNA and protein.

We used a siRNA preparation to reduce PAR1 expression in vitro. A siRNA cocktail directed against PAR1 mRNA markedly reduced PAR1 mRNA and protein; these results were confirmed by real-time qPCR and Western blot analysis (Fig. 5d). Inhibiting PAR1 using siRNA made neurons resistant to granzyme B toxicity. When neurons with inhibited PAR1 expression were chronically exposed to granzyme B, there was no decrease in viability (Fig. 5e). A scrambled siRNA sequence of PAR1 and siRNA for a housekeeping gene (GAPDH) both failed to prevent granzyme B toxicity (Fig. 5e).

### PAR1 activation mediates granzyme B neurotoxicity

**Fig. 6:**
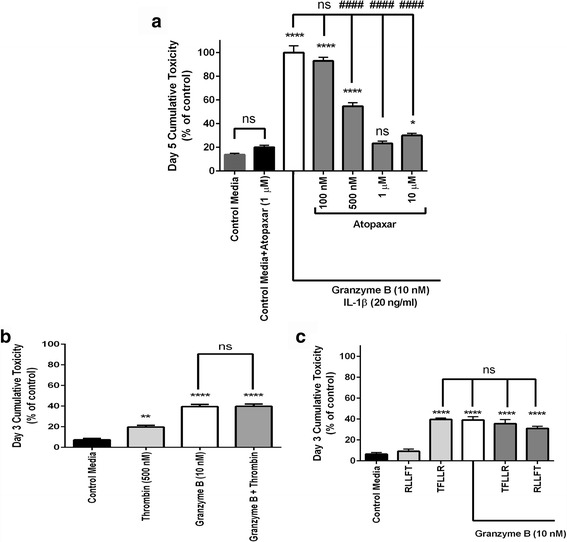
Effects of PAR1 antagonism and agonism. **a** Atopaxar, a selective antagonist of PAR1 significantly reduced granzyme B total cumulative toxicity at 500 nM, 1 μM, and 10 μM pre-treatment doses after 5 days of co-incubation with granzyme B and IL-1β. The cumulative toxicity at the 1 μM dose of Atopaxar was not significantly different from toxicity of control media, which contained the same concentration of DMSO necessary to solubilize Atopaxar, nor was Atopaxar itself significantly more toxic than control media. *p < 0.05, ****p < 0.0001 relative to control media, ####*p* < 0.0001 relative to granzyme B+ IL-1β, repeated measures ANOVA with post hoc Tukey’s test, *n =* 8 per condition. Data are shown as the mean ± standard error of the mean from five separate experiments. **b** Thrombin (500 nM), an agonist of PAR1, causes mild neurotoxicity when added to neuronal cultures for 3 days. When granzyme B is added with thrombin, there is no additive toxic effect. *p < 0.05, ****p < 0.0001 relative to control media, one-way ANOVA with post hoc Tukey’s test, *n =* 10 per condition. Data are shown as the mean ± standard error of the mean from duplicate experiments. **c** The peptide activating sequence TFLLR (5 μM), an agonist of PAR1, reduced neuronal viability after 3 days to the same extent as 10 nM granzyme B. When granzyme B and TFLLR were combined, there was no additional increase in toxicity. RLLFT, the inverse peptide of the agonist sequence, was non-toxic by itself and did not increase toxicity when paired with granzyme B. ****p < 0.0001 relative to control media, one-way ANOVA with post hoc Tukey’s test, *n =* 10 per condition. Data are shown as the mean ± standard error of the mean from three separate experiments

To establish PAR1 as the definitive receptor for the granzyme B-mediated neurotoxicity, two different strategies were attempted, antagonism and the use of alternative PAR agonists. To determine if direct antagonism of PAR1 could prevent neurotoxicity due to granzyme B, an antagonist compound specific to PAR1, Atopaxar (also known as “E5555”), was placed into the media prior to the introduction and replacement of granzyme B. Atopaxar successfully prevented chronic neurotoxicity due to granzyme B in a dose-dependent fashion (Fig. 6a).

To determine if maximum activation of PAR1 had been achieved at the selected dose of granzyme B (10 nM), we added the native ligand for the PAR1 receptor, thrombin, with granzyme B and observed toxicity over the course of 3 days. Thrombin alone was neurotoxic but produced no additional toxicity after pre-treatment with granzyme B (Fig. 6b). As an additional confirmation, the peptide ligand TFLLR-NH_2_, a non-protease agonist of PAR1 receptors, was added with and without granzyme B. This selective agonist proved as toxic as granzyme B when introduced alone, but the inactive reverse sequence of this peptide (RLLFT-NH_2_) did not have any toxic effect (Fig. 6c). When TFLLR-NH_2_ was added with granzyme B, as was the case with thrombin, there was no additional significant increase in toxicity. Therefore, we concluded that the observed toxicity was attributable only to optimized activation of PAR1 and not significantly influenced by off-target effects.

### Inhibition of second messenger systems

Protease activation of PAR1 can trigger several different second messenger entities including phospholipase Cβ, IP3, PI-3-kinase, protein kinase C, and ERK 1/2. We screened several second messenger inhibitors directed against targets within these pathways to investigate whether interference with a specific path could modify the toxicity of chronic granzyme B exposure.

**Fig. 7:**
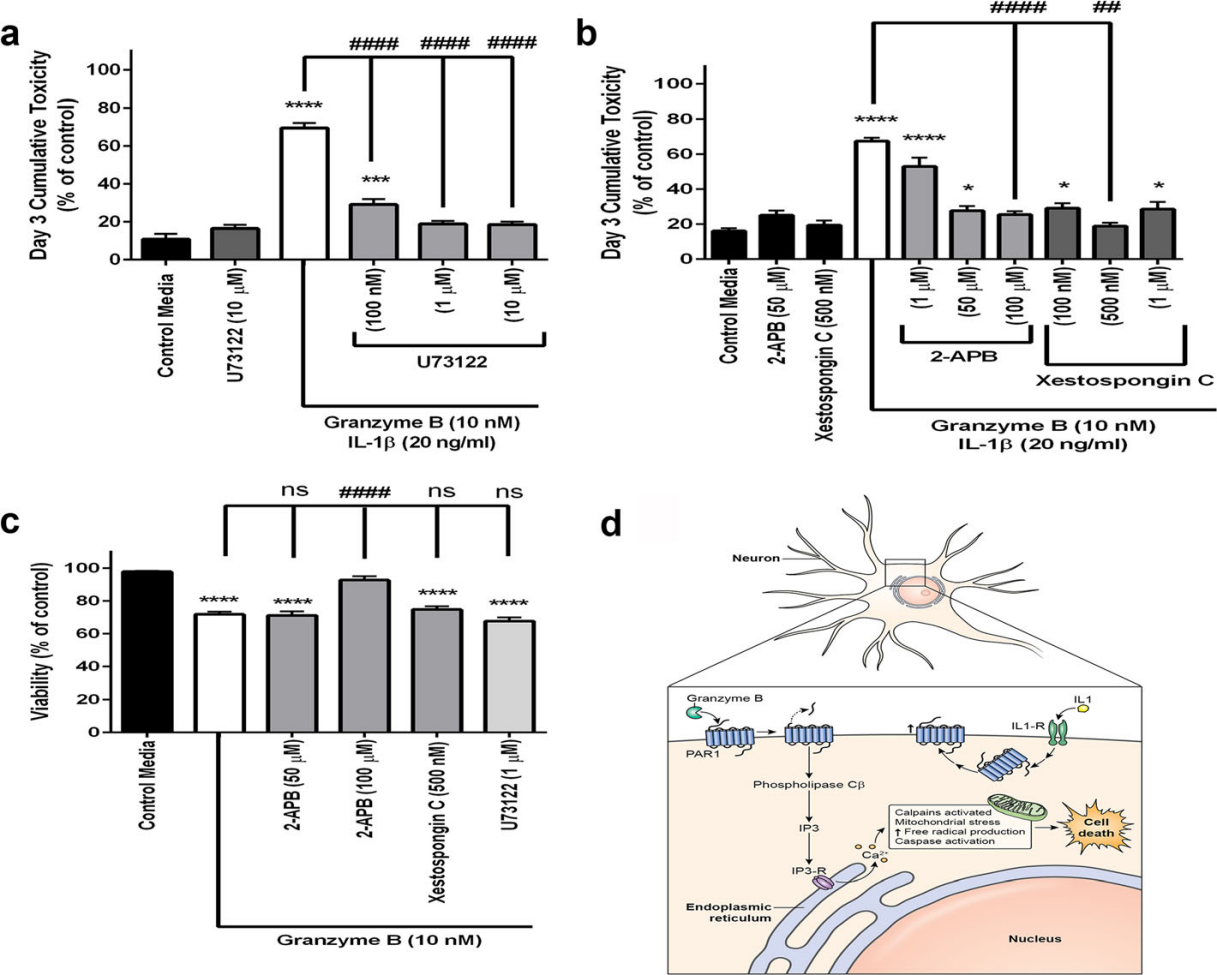
Inhibition of protein kinase C and IP3 reduced neurotoxicity. **a** Pre-treatment with the phospholipase C inhibitor U73122 for 30 min significantly reduced neurotoxicity caused by 3 days of exposure to granzyme B (10 nM) and IL-1β (20 ng/ml). All doses of U73122 were effective at reducing toxicity relative to maximum IL-1β augmented granzyme B-mediated toxicity. The 1 and 10 μM doses reduced toxicity to levels identical to that of control media. ***p < 0.001, ****p < 0.0001 relative to control media with 10% ethanol (vehicle), ####*p* < 0.0001 relative to granzyme B + IL-1β condition, one-way ANOVA with post hoc Tukey’s test, *n =* 5 per condition. Data are shown as the mean ± standard error of the mean from five separate experiments. **b** Several doses of the selective IP3 inhibitors 2-APB and xestospongin significantly reduced the cumulative toxicity typically seen after 3 days. *p < 0.05, ****p < 0.0001 relative to control media and ##*p* < 0.01, ####*p* < 0.0001 relative to granzyme B + IL-1β, one-way ANOVA with post hoc Tukey’s test, *n =* 5 per condition. Data are shown as the mean ± standard error of the mean from four separate experiments. **c** Neuronal viability was preserved in an acute, single day exposure to 10 nM granzyme B following pre-treatment with only 100 μM 2-APB, ****p < 0.0001 relative to control media, ####*p* < 0.0001 relative to granzyme B + IL-1β, one-way ANOVA with post hoc Tukey’s test, *n =* 5 per condition. Data are shown as the mean ± standard error of the mean from duplicate experiments. **d** Proposed mechanism of action for granzyme B-mediated neurotoxicity with IL-1β assistance. IL-1β promotes increased numbers of PAR1 receptors on neuronal membrane surface. Granzyme B cleaves the PAR1 receptors’ extracellular domains. Activated PAR1 directly couples with phospholipase Cβ which in turn yields increased IP3 production and leads to the well-described cellular processes that compromise mitochondrial function and eventually culminate in neuronal demise

We found that blocking phospholipase Cβ hydrolysis of PIP2, an intermediate in the IP3/diacyl glycerol pathway, using the inhibitor U73122 prevented chronic granzyme B-mediated neurotoxicity (Fig. 7a). Inhibition using U73122 had no significant impact on acute granzyme B toxicity as its effect was significant over a time course of several days and evident only when cumulative toxicity was analyzed (Fig. 7c).

The hydrolysis of PIP2 catalyzes the production of inositol trisphosphate (IP3). Two inhibitors of IP3, 2-APB and (−)-xestospongin C, prevented neurotoxicity due to chronic granzyme B (Fig. 7b). The coupling of PAR1 activation through IP3 production completes a clear and known mechanism of neurotoxicity (Fig. 7d). We incidentally observed a dose-dependent improvement in viability following an acute granzyme B toxicity for 2-APB exclusively (Fig. 7c).

**Fig. 8:**
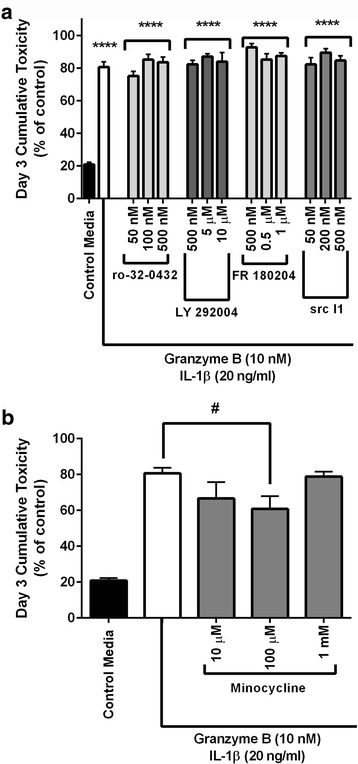
Effect of other second messengers on neurotoxicity. **a** Pre-treatment with four different inhibitors (Ro 32–0432 hydrochloride, LY 294002, FR 180204, Src Inhibitor-1) directed selectively against second messengers (protein kinase C, PI3, ERK, and src kinase, respectively) all of which activated by PAR1 failed to achieve significant reduction in cumulative toxicity after 3 days of repeated exposure to granzyme B and IL-1β. *p < 0.05, ****p < 0.0001 relative to control media, ##*p* < 0.01, ####*p* < 0.0001 relative to granzyme B + IL-1β condition, repeated measures ANOVA with post hoc Tukey’s test, *n =* 6 per condition. Data are shown as the mean ± standard error of the mean from four separate experiments. **b** 100 μM Minocycline produced a modest reduction (<20%) in IL-1β augmented granzyme B neurotoxicity. #*p* < 0.05 relative to granzyme B + IL-1β condition, repeated measures ANOVA with post hoc Tukey’s test, *n =* 5 per condition. Data are shown as the mean ± standard error of the mean from four separate experiments

Pre-treatment with inhibitors targeting protein kinase C (Ro 32–0432 hydrochloride), PI3 (LY 294002), ERK (FR180204), and src kinase (Src Inhibitor-1) had no effect on granzyme B neurotoxicity (Fig. 8a). Minocycline, a known neuroprotective agent, provided a mild reduction in neuronal death due to chronic granzyme B (Fig. 8b).

## Discussion

Chronic granzyme B exposure is neurotoxic because of PAR1 activation. The neurotoxicity mechanism attributed to granzyme B in these experiments occurs via maximal activation of PAR1 on the neuronal surface because adding additional PAR1 agonists to granzyme B did not significantly increase toxicity. Previous research has demonstrated that CSF from patients with MS is directly toxic to cultured rat neurons [29]. The CSF of patients with MS contains abundant granzyme B [18] and IL-1β [19, 30], and CSF IL-1β concentrations correlate with MS-associated brain atrophy [22]. Our research shows that persistent granzyme B drives neurotoxicity and IL-1β augments neuronal death by increasing PAR1 expression. Therefore, the implication of this research is clear: the coincidence of granzyme B and IL-1β within the central nervous systems of patients with neuroinflammation is likely to cause and accelerate neuronal loss. Our finding of reduced total numbers of neurons in brains from patients with MS corroborates prior published immunohistochemistry studies showing the same [31]. The demonstration in this work of PAR1-positive neurons prominently within demyelinated lesions is likewise in keeping with prior reports [32] and confirms that neurons situated within inflamed tissues possess the necessary receptor for granzyme B to activate and are therefore at high risk of loss. It is worth noting that the traditional cytotoxicity of granzyme B is dependent upon co-release of perforin, but perforin-independent neurotoxicity is well-established, and furthermore, this research does not contradict our previous finding of PAR1 cleavage causing downstream activation of the potassium channel Kv1.3 [10, 18]. The observation of IP3 inhibition interfering with acute exposure granzyme B toxicity is likely attributable to a known effect of 2-APB on the Kv1.3 receptor [18].

PAR1 activation by granzyme B can lead to signaling through multiple second messenger pathways. Our research shows activation of the phospholipase-Cβ and PIP2-IP3 pathway is critical to chronic PAR1 neurotoxicity. Phospholipase-Cβ and IP3 have established links to neuroinflammation. An unbiased genome-wide association studies identified a linkage of phospholipase C isoforms to the pathophysiology of MS [33]. IP3 production is increased in neurons at several time points after inoculation against myelin-derived products in the experimental autoimmune encephalitis model of MS suggesting IP3 dysregulation is an early and persistent event in central nervous system inflammation [34]. IP3 is the critical mediator of brain vascular epithelial cell inflammatory response caused by arachidonic acid [35]. Therefore, IP3 is a key player in neuroinflammation, and our work phosopholipase-Cβ and IP3 are significant targets for protecting neurons and glia from toxicity within an inflammatory milieu.

Knowledge of this novel pathological process suggests potential therapeutic considerations. PAR1 contributes to post-stroke neurotoxicity [36], and PAR1 activation causes oligodendrocyte death [37], yet despite experimental data demonstrating thrombin as a neurotoxic agent [38], no trial to date has examined anti-thrombin receptor therapy as a potential treatment for neuroinflammatory-associated neurodegeneration. Several publications have demonstrated that aspirin, an agent that interferes with thrombin (the native agonist of PAR1) reduces fatigue in patients with MS [39, 40]. While the precise mechanism of how aspirin ameliorates MS-related fatigue is not known, the effect is independent of lowering basal body temperature [40] and may be the result of inhibiting IL-1β release. Irreversibly inhibiting thrombin using aspirin would also indirectly decrease PAR1 signaling by decreasing thrombin activity. Activation of the PAR family of receptors on endothelial cells is a major factor in promoting a local inflammatory response that includes cytokine release [41], and PAR inhibitors therefore might be a therapy with manifold clinical benefits in neuroinflammatory diseases. The PAR1 antagonist used in our research, Atopaxar, is presently in human trials for primary prevention of heart attacks and strokes. Atopaxar crosses the blood-brain barrier making it an appropriate medication to consider for neuroprotection [42]. Thus, the use of PAR antagonists such as Atopaxar to prevent neurodegeneration is a credible and unexploited use for this unique class of medications [12].

PAR1 antagonism is only one component of the pathological process we investigated. Studies have shown that higher IL-1β levels in CSF are correlated with progression of clinical disability [43] in MS as well as with MS-related cortical pathology on brain imaging [22]. IL-1β in CSF enhances synaptic excitability [31] which may contribute independently to neuronal dysfunction in the brains of patients with MS. An IL-1 receptor antagonist (anakinra) is an approved therapy for rheumatoid arthritis; therapeutic anakinra levels can be achieved in human CSF [44]. The concentration of endogenous IL-1 receptor antagonists correlates with MS exacerbations in several studies [45, 46]. Medications that antagonize IL-1 receptors therefore have potential as disease-modifying agents in MS both as a means of making relapses less likely and for preventing transition from relapsing-remitting to a progressive form of disease.

Given the failure of single-agent therapies to achieve significant neuroprotection in numerous clinical trials, proposing the concurrent use of two different agents, one to inhibit PAR1 and another to prevent IL-1 receptor signaling, reflects a realization of the complexity of pathology in MS and that promoting effective neuroprotection may require manipulations of multiple pathways. Combination therapy using two agents with differing mechanisms at low doses might ameliorate the potential toxicity of a single agent at a higher dose. Finally, it will be desirable in heterogeneous diseases like MS to have several options to address pathological processes whose relative predominance may vary between patients with the same diagnosis.

The described studies have limitations. The IL-1β and granzyme B concentrations we detected in the CSF of patients with progressive MS were within the ranges noted by others who have investigated CSF samples from patients with MS [18]. It would be expected that using these methods with CSF samples from patients diagnosed with the most common presentation of MS, relapsing-remitting, would replicate and expand our neurotoxicity findings.

While the combination of granzyme B and IL-1β demonstrated considerable toxicity in vitro, there are certainly many other potential factors that could impact neurotoxicity in vivo. Our work and that of others [47] suggests that IL-1β augments other toxins’ potencies, but our findings do not infringe on the certainty that there are other primary and secondary agents (both cellular and soluble) that contribute to the complex pathology of MS.

Though there is a published correlation between IL-1β in CSF and brain atrophy [22], no published studies have correlated granzyme B levels with clinical or radiographic measures of pathology in progressive forms of MS. Because of the anonymity of the donated human samples, we were unable to correlate the in vitro findings with any clinical parameters. Future investigations should attempt to correlate brain volumetric measurements with CSF levels of granzyme B together with IL-1β in the same patients longitudinally to see if high levels of these inflammatory entities are as synergistic in vivo as they appear to be in vitro. It would be worthwhile to see if clinical correlations exist between clinical parameters such as cognitive assessments or fatigue ratings and CSF granzyme B or IL-1β levels.

Since these studies were done in single-cell cultures, this model is not a true replication of the central nervous system environment which features multiple coexisting neuronal and glial cell populations. Investigators have shown widespread PAR1 expression in several cell types within the brain [8, 13, 15], and there is evidence that granzyme B and IL-1β are each independently toxic to glia [47, 48]. Future investigations should examine whether these inflammatory mediators together might provide another mechanism for glial or endothelial toxicity in MS. Brain atrophy related to MS might occur indirectly through accelerated glial loss or enhanced neurotoxicity through disruption of the neuronal microenvironment.

The mechanism of how IL-1β upregulates PAR1 was not definitively shown in this research. While the IL-1 receptor signals through protein kinase C, we did not see any change in IL-1β toxicity with two different protein kinase C inhibitors. Since direct antagonism of the IL-1 receptor fully inhibited the IL-1β effect, we do not believe that the observed toxicity increase is receptor independent and speculate that another entity linked to the IL-1 receptor such as calmodulin or NF-κB might be potential mediators of the effect.

The use of *E. coli*-derived products is a potential concern because recombinant products produced in other species have glycosylation that differs from native human substances and potentially different effects than their human analogues. However, the total replication of the recombinant products’ in vitro neurotoxicity using human CSF, and the correlation between CSF levels of granzyme B and IL-1β in this research suggests that the recombinant products were adequate to demonstrate the same effects as human-derived compounds. While this research used a pure population of human neurons (predominantly glutamatergic and GABAergic), this is a novel strength of this work since other publications have used rodent (not human) neurons as targets for human-derived reagents.

While our preparation was specifically designed to exclude immune cells from the experiment and isolate any findings to just soluble factors, we acknowledge that an extensive literature exists concerning the roles of lymphocytes such as activated T cells and natural killer cells, the sources of granzyme B and IL-1β, respectively, in the pathology of progressive MS [49, 50]. Certainly, lymphocyte activation precedes granzyme B and IL-1β effects, but our research supports the conclusion that ongoing exposure to these two soluble immune factors is sufficient to create an adverse environment for neurons exposed to inflammatory CSF.

## Conclusions

Our findings elucidate a novel mechanism for how chronic exposure to IL-1β increases neuronal vulnerability to granzyme B toxicity through activation of PAR1. Despite having plausible roles in central nervous system inflammation, these moieties and their downstream effectors have not been targeted specifically by current therapeutic strategies used in the treatment of neuroinflammatory diseases. It is our hope that this report will spur investigation into possible interventions for brain volume loss which has proven to date to be an intractable process in neuroinflammatory diseases.

## Abbreviations

CSF: Cerebrospinal fluid
DAPI: 4′,6-Diamidino-2-phenylindole
IL-1: Interleukin-1
IL-1β: Interleukin-1β
MS: Multiple sclerosis
PAR1: Protease-activated receptor-1

## References

[1] Anderson DW, Ellenberg JH, Leventhal CM (1992). Revised estimate of the prevalence of multiple sclerosis in the United States. Ann Neurol..

[2] Rissanen E, Tuisku J, Rokka J (2014). In Vivo Detection of Diffuse Inflammation in Secondary Progressive Multiple Sclerosis Using PET Imaging and the Radioligand 11C-PK11195. J Nucl Med..

[3] Popescu V, Agosta F, Hulst HE (2013). Brain atrophy and lesion load predict long term disability in multiple sclerosis. J Neurol Neurosurg Psychiatry..

[4] Fernández-Jaén A, Fernández-Mayoralas DM, Fernández-Perrone AL (2014). Cortical thickness at the time of the initial attack in two patients with paediatric relapsing-remitting multiple sclerosis. Eur J Paediatr Neurol..

[5] De Stefano N, Giorgio A, Battaglini M (2010). Assessing brain atrophy rates in a large population of untreated multiple sclerosis subtypes. Neurology.

[6] Calabrese M, Poretto V, Favaretto A (2012). Cortical lesion load associates with progression of disability in multiple sclerosis. Brain..

[7] Klaver R, De Vries HE, Schenk GJ, Geurts JJ (2013). Grey matter damage in multiple sclerosis: a pathology perspective. Prion..

[8] Wang H, Reiser G (2003). Thrombin signaling in the brain: the role of protease-activated receptors. Biol Chem..

[9] Vu TK, Hung DT, Wheaton VI, Coughlin SR (1991). Molecular cloning of a functional thrombin receptor reveals a novel proteolytic mechanism of receptor activation. Cell..

[10] Wang T, Lee MH, Choi E (2012). Granzyme B-induced neurotoxicity is mediated via activation of PAR-1 receptor and Kv1.3 channel. PLoS One.

[11] Scarisbrick IA, Isackson PJ, Ciric B, Windebank AJ, Rodriguez M (2001). MSP, a trypsin-like serine protease, is abundantly expressed in the human nervous system. J Comp Neurol..

[12] Ramachandran R, Noorbakhsh F, Defea K, Hollenberg MD (2012). Targeting proteinase-activated receptors: therapeutic potential and challenges. Nat Rev Drug Discov..

[13] Olianas MC, Dedoni S, Onali P (2007). Proteinase-activated receptors 1 and 2 in rat olfactory system: layer-specific regulation of multiple signaling pathways in the main olfactory bulb and induction of neurite retraction in olfactory sensory neurons. Neuroscience..

[14] Olson EE, Lyuboslavsky P, Traynelis SF, McKeon RJ (2004). PAR-1 deficiency protects against neuronal damage and neurologic deficits after unilateral cerebral hypoxia/ischemia. J Cereb Blood Flow Metab..

[15] Almonte AG, Qadri LH, Sultan FA, Watson JA, Mount DJ, Rumbaugh G, Sweatt JD (2013). Protease-activated receptor-1 modulates hippocampal memory formation and synaptic plasticity. J Neurochem..

[16] Acharjee S, Zhu Y, Maingat F (2011). Proteinase-activated receptor-1 mediates dorsal root ganglion neuronal degeneration in HIV/AIDS. Brain..

[17] Donovan FM, Pike CJ, Cotman CW, Cunningham DD (1997). Thrombin induces apoptosis in cultured neurons and astrocytes via a pathway requiring tyrosine kinase and RhoA activities. J Neurosci..

[18] Wang T, Lee MH, Johnson T (2010). Activated T-cells inhibit neurogenesis by releasing granzyme B: rescue by Kv1.3 blockers. J Neurosci..

[19] Sellebjerg F, Bendtzen K, Christiansen M, Frederiksen J (1997). Cytokines and soluble IL-4 in patients with acute optic neuritis and multiple sclerosis. Eur J Neurol..

[20] Mehta PD, Kulczycki J, Mehta SP (1997). Increased levels of interleukin-1beta and soluble intercellular adhesion molecule-1 in cerebrospinal fluid of patients with subacute sclerosing panencephalitis. J Infect Dis..

[21] Rossi S, Furlan R, De Chiara V (2012). Interleukin-1β causes synaptic hyperexcitability in multiple sclerosis. Ann Neurol..

[22] Seppi D, Puthenparampil M, Federle L (2014). Cerebrospinal fluid IL-1β correlates with cortical pathology load in multiple sclerosis at clinical onset. J Neuroimmunol..

[23] Takahashi Y, Mine J, Kubota Y, Yamazaki E, Fujiwara T (2009). A substantial number of Rasmussen syndrome patients have increased IgG, CD4+ T cells, TNFalpha, and Granzyme B in CSF. Epilepsia..

[24] Gnanapavan S, Grant D, Morant S (2013). Biomarker report from the phase II lamotrigine trial in secondary progressive MS - neurofilament as a surrogate of disease progression. PLoS One.

[25] Jia Y, Wu T, Jelinek CA (2012). Development of protein biomarkers in cerebrospinal fluid for secondary progressive multiple sclerosis using selected reaction monitoring mass spectrometry (SRM-MS). Clin Proteomics.

[26] Shimormura N, Sasho M, Kayano A, inventors; Esai Co., Ltd., assignee (2004). Methods for producing cyclic benzamidine derivatives.

[27] Hedreen JC (1998). What was wrong with the Abercrombie and empirical cell counting methods? A review. Anat Rec..

[28] Bae M, Patel N, Xu H (2014). Activation of TRPML1 clears intraneuronal Aβ in preclinical models of HIV infection. J Neurosci..

[29] Wang T, Allie R, Conant K (2006). Granzyme B mediates neurotoxicity through a G-protein-coupled receptor. FASEB J..

[30] Cacabelos R, Barquero M, García P (1991). Cerebrospinal fluid interleukin-1 beta (IL-1 beta) in Alzheimer's disease and neurological disorders. Methods Find Exp Clin Pharmacol..

[31] Vercellino M, Plano F, Votta B (2005). Grey matter pathology in multiple sclerosis. J Neuropathol Exp Neurol..

[32] Junge CE, Lee CJ, Hubbard KB (2004). Protease-activated receptor-1 in human brain: localization and functional expression in astrocytes. Exp Neurol..

[33] Bush WS, McCauley JL, DeJager PL (2011). A knowledge-driven interaction analysis reveals potential neurodegenerative mechanism of multiple sclerosis susceptibility. Genes Immun..

[34] Di Prisco S, Merega E, Milanese M (2013). CCL5-glutamate interaction in central nervous system: Early and acute presynaptic defects in EAE mice. Neuropharmacology..

[35] Evans J, Ko Y, Mata W (2015). Arachidonic acid induces brain endothelial cell apoptosis via p38-MAPK and intracellular calcium signaling. Microvasc Res..

[36] Xue M, Hollenberg MD, Demchuk A, Yong VW (2009). Relative importance of proteinase-activated receptor-1 versus matrix metalloproteinases in intracerebral hemorrhage-mediated neurotoxicity in mice. Stroke..

[37] Burda JE, Radulovic M, Yoon H, Scarisbrick IA (2013). Critical role for PAR-1 in kallikrein 6-mediated oligodendrogliopathy. Glia..

[38] da Lee Y, Park KW, Jin BK (2006). Thrombin induces neurodegeneration and microglial activation in the cortex in vivo and in vitro: proteolytic and non-proteolytic actions. Biochem Biophys Res Commun..

[39] Shaygannejad V, Janghorbani M, Ashtari F, Zakeri H (2012). Comparison of the effect of aspirin and amantadine for the treatment of fatigue in multiple sclerosis: a randomized, blinded, crossover study. Neurol Res.

[40] Wingerchuk DM, Benarroch EE, O'Brien PC (2005). A randomized controlled crossover trial of aspirin for fatigue in multiple sclerosis. Neurology..

[41] Alberelli MA, De Candia E (2014). Functional role of protease activated receptors in vascular biology. Vascul Pharmacol..

[42] Kai Y, Hirano K, Maeda Y (2007). Prevention of the hypercontractile response to thrombin by proteinase-activated receptor-1 antagonist in subarachnoid hemorrhage. Stroke..

[43] Rossi S, Studer V, Motta C (2014). Cerebrospinal fluid detection of interleukin-1β in phase of remission predicts disease progression in multiple sclerosis. J Neuroinflammation..

[44] Galea J, Ogungbenro K, Hulme S (2011). Intravenous anakinra can achieve experimentally effective concentrations in the central nervous system within a therapeutic time window: results of a dose-ranging study. J Cereb Blood Flow Metab..

[45] Dujmovic I, Mangano K, Pekmezovic T (2009). The analysis of IL-1 beta and its naturally occurring inhibitors in multiple sclerosis: The elevation of IL-1 receptor antagonist and IL-1 receptor type II after steroid therapy. J Neuroimmunol..

[46] Voltz R, Hartmann M, Spuler S (1997). Multiple sclerosis: longitudinal measurement of interleukin-1 receptor antagonist. J Neurol Neurosurg Psychiatry..

[47] Takahashi JL, Giuliani F, Power C (2003). Interleukin-1beta promotes oligodendrocyte death through glutamate excitotoxicity. Ann Neurol..

[48] Kroner A, Ip CW, Thalhammer J (2010). Ectopic T-cell specificity and absence of perforin and granzyme B alleviate neural damage in oligodendrocyte mutant mice. Am J Pathol..

[49] Romme Christensen J, Börnsen L, Ratzer R (2013). Systemic inflammation in progressive multiple sclerosis involves follicular T-helper, Th17- and activated B-cells and correlates with progression. PLoS One..

[50] De Biasi S, Simone AM, Nasi M (2016). iNKT Cells in Secondary Progressive Multiple Sclerosis Patients Display Pro-inflammatory Profiles. Front Immunol..

